# Wearables for Biomechanical Performance Optimization and Risk Assessment in Industrial and Sports Applications

**DOI:** 10.3390/bioengineering9010033

**Published:** 2022-01-13

**Authors:** Sam McDevitt, Haley Hernandez, Jamison Hicks, Russell Lowell, Hamza Bentahaikt, Reuben Burch, John Ball, Harish Chander, Charles Freeman, Courtney Taylor, Brock Anderson

**Affiliations:** 1 Department of Electrical & Computer Engineering, Mississippi State University, Starkville, MS 39765, USA; sam1123@msstate.edu (S.M.); hnk50@msstate.edu (H.H.); jeball@ece.msstate.edu (J.B.); 2 Department of Industrial & Systems Engineering, Mississippi State University, Starkville, MS 39765, USA; jsh614@msstate.edu (J.H.); burch@ise.msstate.edu (R.B.); 3 Neuromechanics Laboratory, Department of Kinesiology, Mississippi State University, Starkville, MS 39765, USA; rkl84@msstate.edu (R.L.); hchander@colled.msstate.edu (H.C.); 4 Department of Mechanical Engineering, Mississippi State University, Starkville, MS 39765, USA; hb1142@msstate.edu; 5 Human Factors & Athlete Engineering, Center for Advanced Vehicular Systems, Mississippi State University, Starkville, MS 39765, USA; 6 Department of Human Sciences, Mississippi State University, Starkville, MS 39765, USA; 7 Accelerate MS, Jackson, MS 39201, USA; courtneytaylor@acceleratems.org; 8 Ergo-Ology, Atlanta, GA 31139, USA; info@ergo-ology.com

**Keywords:** wearables, biomechanics, risk assessment, performance optimization, athletics, wearable ergonomics, exoskeleton

## Abstract

Wearable technologies are emerging as a useful tool with many different applications. While these devices are worn on the human body and can capture numerous data types, this literature review focuses specifically on wearable use for performance enhancement and risk assessment in industrial- and sports-related biomechanical applications. Wearable devices such as exoskeletons, inertial measurement units (IMUs), force sensors, and surface electromyography (EMG) were identified as key technologies that can be used to aid health and safety professionals, ergonomists, and human factors practitioners improve user performance and monitor risk. IMU-based solutions were the most used wearable types in both sectors. Industry largely used biomechanical wearables to assess tasks and risks wholistically, which sports often considered the individual components of movement and performance. Availability, cost, and adoption remain common limitation issues across both sports and industrial applications.

## 1. Introduction

Wearable technologies are a growing are of interest due to their potential benefits for biological feedback data collection through non-invasive monitoring of users. These devices can be used in several biofeedback applications, including: physiological (e.g., heart rate), neurological (e.g., brain-wave), biochemical (e.g., metabolites), and bio-mechanical (e.g., joint angles; [1]). As such, the use of wearables continues to increase across several populations, including athletic, recreational sporting, occupational, clinical, geriatric, pediatric, and daily living. However, wearables in athletics and industry have increased in demand and function over recent years.

When considering worker performance, industry workers that engage in physical activities are often considered “industrial athletes” [2,3]. These are individuals who experience repetitive motion tasks in occupational settings such as in manufacturing, warehousing, logistics, and other service industries. In these cases, industrial athletic concerns often overlap with the more commonly associated issues experienced by the “sports athlete” [4]. Given how quickly the landscape of wearables technologies changes, the purpose of this article is to provide a focused narrative literature review of recent biomechanical wearable device studies. This literature review highlights studies that demonstrate the use of wearable device use in performance optimization and risk assessments associated with both industries (e.g., material handling, manufacturing) and athletics (i.e., sports).

### Wearables for Biomechanical and Risk Assessments

Biomechanical wearable devices consist of various types of technology to augment performance as well as to assess human performance during tasks and movements. The use of these devices can assist health and safety professionals, ergonomists, and human factors practitioners to optimize both industrial and sports athlete performance while identifying potential biomechanical injury risks and mitigating those risks by utilizing data driven decision making from information collected while using the wearable devices [5]. Performance enhancement devices that are often related to the augmentation of hu-man performance consist of exoskeletons, micro-electromechanical systems such as inertial measurement units (IMU’s), and networked sensor suites [6,7]. Risk assessment wearables often overlap with performance enhancement devices, and the data collected can be used to enhance the understanding of performance while assessing the risk of injury. Risk assessment wearables include pressure sensors [8] and IMU’s that host several sensors (e.g., accelerometers, gyroscopes, and magnetometers; [9]).

With such increased demand and function of wearable technology, especially in industry and sports [10], this literature review intends to provide health and safety professionals, ergonomists, and human factors practitioners with an overview of the biomechanical applications of wearable technologies and their associations, focusing on industry and sports applications.

## 2. Materials and Methods

**Figure 1:**
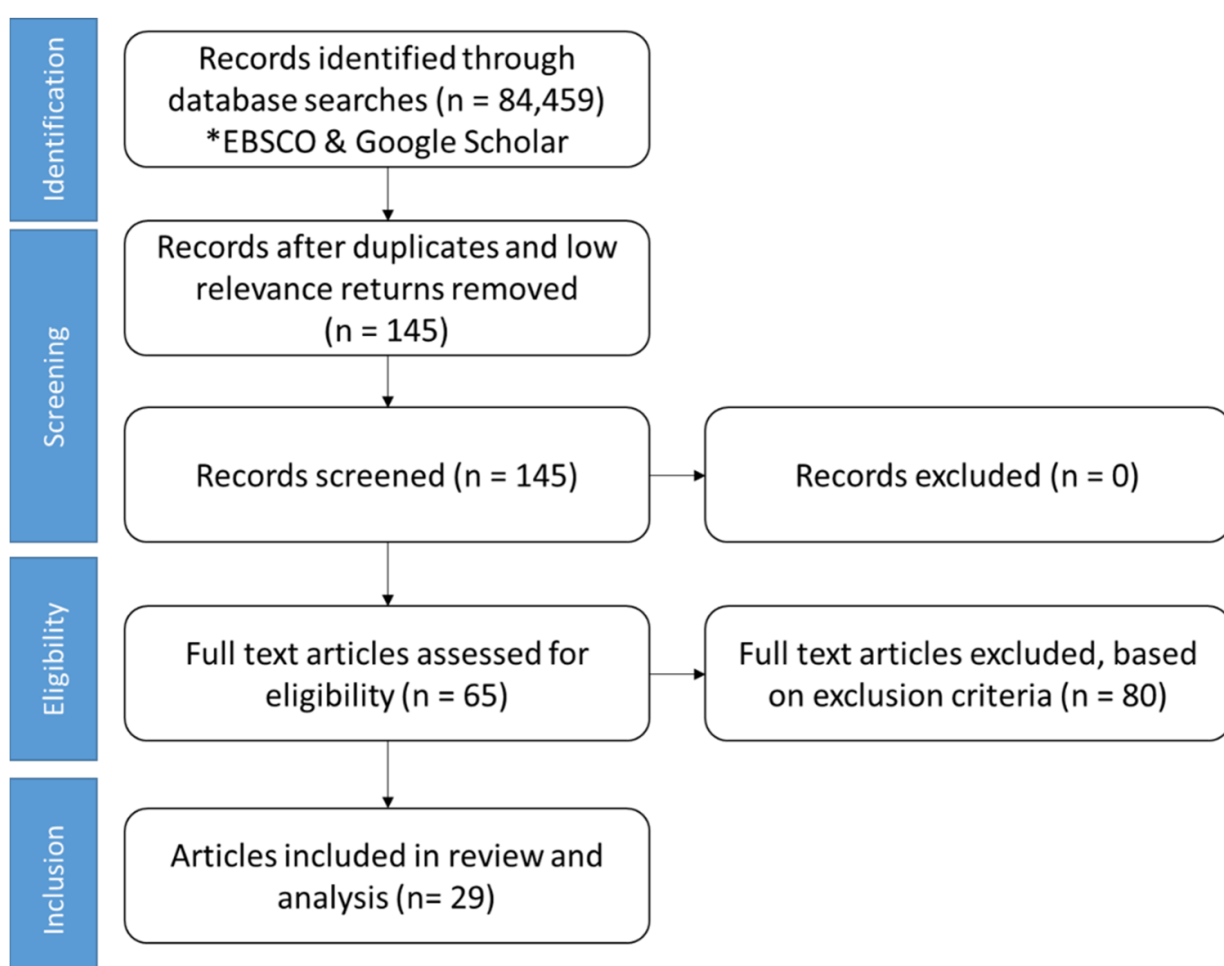
Strategy for literature search using PRISMA guidelines. * Based on literature review criteria search engines.

This literature review surveyed wearable applications across industries (e.g., manufacturing, manual task labor, and automated environments) and sports to highlight select contributions of wearable technologies related to biomechanical performance optimization and risk assessment. A review of academic research databases (i.e., Google Scholar, EBSCO) using the PRISMA guidelines is provided in Figure 1.

The methods used to identify a research question, search strategy and article selection along with the data extraction, analysis and results and reporting of the identified findings are presented in both Table 1 and Figure S1.

A Microsoft Excel^TM^ spreadsheet was used to capture search criteria (e.g., databases searched, constraints, articles reviewed, saved, rejected, information summary, and relevance to the research questions). After initial abstract reviews, the following criteria determined the selection of articles for further review: (1) relevance to the research question, (2) experimental data reporting the use of wearable technologies for biomechanical performance enhancement or risk assessment, or (3) a literature review of other biomechanical related wearable research.

Database searches returned over 60,000 keyword matches, but most only included some of the keywords without direct relation to the research question. Research publications were primarily considered from the years 2015–2021 to identify the most recent technological developments. Based on the initial reviews, sixty-five (65) articles identified as relevant to the research questions with twenty-nine (29) articles fitting into the inclusion criteria as the research addressed the two questions. The remaining articles did not contain enough information pertinent to the research questions for inclusion or were similar to the chosen articles. This literature review is not intended to be an exhaustive canvas of all wearable applications given the quickly changing landscape of use and broad user application. However, the selected papers show the breadth of application of wearable technologies to address the research questions and to provide a guide for health and safety decision-making practitioners servicing both the sports and industrial athletes.

## 3. Results

### 3.1. Wearables in Biomechanical Performance Optimization

#### 3.1.1. Industrial Athlete Performance Biomechanical Applications

Wearable devices are much more common in biomechanical use cases for the sports athlete and fitness applications than for industrial athlete applications [11], primarily due to technological, economic, and worker privacy concerns [12,13]. However, significant efforts are underway to improve wearable device functionality and industrial applications for monitoring both worker safety and performance [14]. Biomechanical wearables for performance enhancement in industrial applications fall into two primary categories: (1) assisting and (2) monitoring [12]. Exoskeleton augmentations heavily dominate assistive devices.

Exoskeletons are wearable machine devices that augment human performance, primarily for heavy lifting tasks [15]. Industrial exoskeletons have been developed and analyzed in numerous studies to augment human performance and reduce the likelihood of injury. For example, a survey by Sylla et al. (2019) adapted a rehabilitation exoskeleton for automotive manufacturing tasks that required significant upper body movement. Results indicated that an exoskeleton significantly reduced the required mechanical energy for tasks, which can help improve worker performance by reducing fatigue [6]. Based on a series of exoskeleton-based studies, researchers have demonstrated that this assistive-based technology improves both range of motion [16,17] and muscle fatigue or activation [16,18,19,20,21,22,23,24,25,26,27,28,29,30,31,32,33,34,35,36,37,38,39,40,41].

In one study based around range of motion, Spada et al. (2017) utilized an upper limb exoskeleton for static lifting tasks with performance increases identified in load handling of up to 30% [38]. In another study regarding muscle activation, Blanco et al. (2018) utilized electromyography (EMG) to assess how much muscular load was removed from the upper body while wearing an exoskeleton during an overhead drilling task. Significant reductions in activations for both the pectoralis and rhomboids were found when using an exoskeleton while no significant difference was found in the triceps brachii [27]. While exoskeletons can improve performance, current limitations, such as cost, usability, and potential injury, are valid concerns that companies presently have for exoskeleton use in industry [42]. Advances in exoskeleton design are currently under development, with significant research devoted to computer models to optimize designs that safely enhance muscle performance without impeding natural motion [15,43,44].

Industrial applications of monitoring biomechanical performance primarily consist of wearable devices to characterize worker tasks (real-time and post-processing) and adjusting the tasks to optimize performance and ensure worker safety [45]. Significant efforts have been made to utilize IMU devices (e.g., accelerometers, gyroscopes, magnetometers) to characterize worker posture and activities for modeling, performance analysis, exposure analysis, and task redesign [45]. As IMU devices continue to miniaturize, their use and acceptance in industrial monitoring applications will likely increase—just as they have proven to be the most critical technology in sports wearables [24] as of the writing of this literature review—allowing for real-time monitoring of worker performance and identifying potential issues.

“Operator 4.0” is a recent concept in industry design that conceptualizes the future workplace and places the industrial athlete into a networked environment of systems, including significant cooperation with automation and robotic processes [7] as well as an indoor localization system to monitor are sensors donned by workers [46]. As part of Operator 4.0, wearable sensors are predicted play a significant role in the performance optimization of operators through smart sensors that enhance ergonomic best practices through real-time monitoring of operator posture and physical workload [7]).

As wearable technologies for industry advance in the realm of biomechanical task assessment, these systems will need to communicate through a layered data structure that integrates collected data throughout the workers’ environment to optimize performance and ensure worker health as a holistic system [46]. As smart factories continue to develop, advancements in industrial wearable devices will play an essential role in optimizing worker performance through those task components previously mentioned such as range of motion [16,17] and muscle fatigue or activation [16,18,19,20,21,22,23,24,25,26,27,28,29,30,31,32,33,34,35,36,37,38,39,40,41].

#### 3.1.2. Sports Athlete Performance Biomechanical Applications

Regardless of recent applications, health and safety decision makers are looking to expand industry applications for biomechanical wearables [9] and their implementation effectiveness seen in the sports sector. Athletes and their performance staff were some of the first user groups to adopt this technology [47,48] and are constantly seeking innovative methods to improve athletic performance through biomechanical data collections [49]. With the improvement of wearable technology and micro-electromechanical systems, wearable devices have been sought out to improve athletic performance [49,50]. Zhang et al. (2019) states, “It is already a universal agreement that wearable technology is guiding a revolution in sports [1].” The goal of these wearable devices is to monitor sports athletes’ physical condition, mostly through their movements and loading, to mitigate injuries. Thus, real-time biofeedback has the potential to allow coaches to alter their training regimen, if necessary, to reduce injury risk [1]. The capabilities of these devices can span from hydration and metabolism monitoring to physical biofeedback and sleep [51]. With the most common wearable devices being heart/pulse rate monitors and sleep trackers, some literature would say there is a lack of wearable devices that monitor and track real-time biomechanical feedback in sports [1]. Some researchers state this is due to biomechanical feedback needing to be tailored to an activity; the wearable device’s non-generalized motor skills parameters need to be understood [1]. Previous studies have measured biomechanical differences during sporting motions using IMU devices despite the obstacles for real-time biomechanical feedback. There are biomechanical-tracking options available. However, they may be both too expensive and invasive to wear for more basic training regimens with athletes who are not competing at elite levels [47,48,52]) and indeed may provide a level of detail beyond what the wearer needs.

A study performed with male soccer team participants in Spain utilized accelerometers to observe multi-joint external workload profiles during different speeds on a treadmill [53]. Gómez-Carmona et al. (2021) found that the highest tri-axial accelerometry-based workload, or player load, was found at the lower limb and especially at the foot–ankle complex which corroborates the feedback from strength and conditioning coaches interviewed across the United States [47,48,53]. Soccer is a common sport task for assessment with IMUs as another European study with elite athletes’ measured running kinetics. Researchers also using a treadmill, Hughes et al. (2020) captured initial peak acceleration (IPA) and IPA-symmetry index measurement during running [54]. They used two different quality IMUs and found that only the laboratory gold-standard validated, research-oriented IMU was able to demonstrate acceptable minimal detectable changes in IPA [54]. Another soccer study used an IMU-based system, the Xsens MVN^TM^ system, to quantify kicking biomechanics against gold standard Vicon^TM^ motion cameras. Blair et al. (2018) found that lower extremity and pelvis kinematics only had mean differences of 0.2–5.8% between the Xsens MVN^TM^ and Vicon^TM^ [55]. The results of these previous two studies are important to demonstrate that IMU data can be very accurate but that the research-oriented solutions are often more expensive and may be more costly than sport practitioners are able to afford [48].

In a study performed by Shi et al. (2020), a sensor-based analysis was used to observe significant kinematic differences during figure skating jumps with variant revolutions. With the use of five IMU devices attached to the posterior side of the body, the authors were seeking to analyze and compare kinematic differences of the jump to previous studies (video-based methods; [56]). In this study, one nationally ranked competitive male figure skater participated and performed single, double, and triple flip jumps. The results showed that the IMU devices were able to determine significant differences between take-off and flight time compared to video-based methods [56].

Baseball pitchers have the capacity of rotating their throwing arms at 7000°/s [57]. Current methods of capturing movement at such high speeds while maintaining data accuracy require access to a laboratory environment and equipment with high sampling abilities [57]. A proposed method addressed the challenges of quantifying high-speed pitching. The authors created a new IMU device that could capture three-dimensional (3D) motion at low and high speeds. The term “jerk” was introduced as a concept of evaluating pitching mechanics with the IMU system. Lapinski et al. (2020) defined jerk as a rate of change of acceleration. Players pitched a minimum of 25 pitches with a mix of fastballs, breaking balls, and changeups with five IMU devices placed on the wrist, forearm, upper arm, chest, and waist. When compared to 3D motion capture, the results showed that IMU devices were able to capture the peak of the motion during the compression force phase, which is considered the most critical phase of pitching [57]. Peak jerk results occurred between times of peak acceleration, signifying an assessment of the moment at which the peak jerk occurs could be relevant [57].

### 3.2. Wearables in Biomechanical Risk Assessment

While performance optimization holds a valuable place in the wearable market, another key feature for these devices is risk assessment. This task can be achieved by continuously monitoring an individual for crucial data points and providing an early warning to avoid a potential injury. Applications for this technology exist in both the sports domain as well and the workplace.

#### 3.2.1. Industrial Athlete Risk Assessment Applications

According to the 2019 report from the Bureau of Labor Statistics (BLS), over 2.8 million nonfatal workplace injuries and illnesses occurred, with some of the leading industries being construction, manufacturing, and healthcare [58]. In construction, falls account for a significant portion of injuries and even deaths [59]. Repetitive movements in manufacturing increase the likeliness of ergonomic injuries [60]. Given the many different hazards presented in various occupations, wearable technology holds the potential to mitigate injuries using risk assessment.

For the construction worker industrial athlete, there are many safety hazards on the job sites, and the hazards can vary based on the type of project. One threat that is prevalent throughout most scenarios is a fall on the same level. Essentially, these falls occur when a worker loses his or her balance. A research team identified these incidents could be linked to biomechanical gait stability parameters [59]. The researchers utilized IMU-based sensors to track balance loss while simultaneously recording biomechanical parameters from insole pressure systems. This study concluded that there were significant differences in the gait that could be observed in an individual engaging in different types of loss of balance events. Furthermore, the results provide the grounds to develop alternative insole monitoring systems that can allow construction managers to track these events and identify risks that can be eliminated on the job site [59].

Another area of interest when utilizing wearable technology to capture human performance is quantifying ergonomic risk in manufacturing environments where repetitive motions are a concern. Ergonomic risk has been traditionally observed using subjective observational methods or manual measurements performed by an assessor [61]. These types of surveys are subjective, based on the individual filling out the forms, so wearable technology seeks to standardize this method. One group of researchers designed a device composed of nine IMU sensors to simulate the National Institute for Occupational Safety and Health (NIOSH) Lifting Equation, a standard formula in ergonomics that determines safe lifting practices for an employee [60]. In this experiment, the researchers compared the score determined by the wearable system to the manually calculated score. Although the research team advised that future work will be necessary to increase the accuracy of their system, the project did provide evidence that this sort of wearable device could be possible.

#### 3.2.2. Sports Athlete Risk Assessment Applications

Just as in industrial applications, sports athletes are using wearables for biomechanical risk assessment has shown positive impact in the sports world [62]. Wearables have many uses for assessing the risk in how an athlete moves, trains, or recovers. Running is an activity that most athletes do to train, and there are many wearable solutions—such as instrumented insoles—to measure ground reaction forces (GRF) of each footfall. Matijevic et al. (2020) performed a study using IMUs, pressure-sensing insoles, and machine learning to quantify peak tibial force [9]. One of the leading causes of injuries for runners and athletes is tibial overuse. The authors compared IMU’s, pressure-sensing insoles, and Global Positioning System (GPS) data to measure foot rotation angles, GRF, speed, and slope. The result was a two-sensor system that could provide tibial force estimates better than GRF methods and a system that could apply for creating different wearable devices for measuring strain on musculoskeletal structures during activity [9]. Through studies such as these, bone impact and lower leg symmetry data is now available through IMU products such as IMeasureU^TM^ which have been validated against laboratory gold standard Vicon 3D motion capture cameras. IMeasureU^TM^ sensors have been used to assess impact load metrics during soccer-specific performance tasks [63]. These products provide a new tool to measure symmetries in critical baselining metrics such jump studies (Burch et al., 2019). One study used IMeasureU to differences in peak knee flexion and flexion displacement during jump studies [64] which can be used by practitioners to modify workout regimens for mitigation of potential injury.

A study by Powell et al. (2020) focused on pitchside gain and balance analysis to determine if a rugby player has had a concussion. A wearable sensor provided a method for concussion detection using gait analysis that could be used in several sports as a risk assessment technique [65]. A similar study by Grafton et al. (2019) used a head mounted IMU to detect balance impairments [66]. The resulting difference in sway could be helpful information to remove a player from the activity and decrease the overall risk of recurring concussions. Further, similar studies in American football that use wearable sensors for concussion identification further translate the biomechanical findings into feedback regarding helmet and other protective equipment improvement recommendations [67].

Another risk assessment factor in sport is baselining sports athletes’ performance [68] to measure the athlete’s ability to return to sport after an injury. Anterior cruciate ligament (ACL) injuries have a low return to sport rate, and a high graft reinjury rate. Participants in a study that reassessed athletes two years after their ACL reconstruction demonstrated that all athletes had some functional deficit in at least one of their biomechanical movement tests [69]. This demonstrates the criticality of being able to track player return-to-play metrics after the formal rehabilitation has ended. In a study by Dan et al. (2019), researchers use the ViMove wearable sensor and a Matscan pressure mat to observe a return to sports performance. After an ACL reconstruction, the researchers found that the athlete had more varus/valgus movement in the knee when compared to a healthy athlete. The pressure sensor also recorded that the athlete would put more weight on the healthy leg when performing double leg activity. The result was an assessment tool that directed slight alignment differences that could mitigate the risk of reinjury [70]. Further work performed in aiding practitioner understanding of ACL injury mitigation used two IMU systems, MTw Awinda^TM^ and Xsens^TM^, to assess motor coordination and lower limb biomechanics in young soccer players. Research found significant asymmetries were found in the poorly coordinated group of players where “poor motor coordination elicited altered hip and knee biomechanics during sport-specific dynamic movements. [71]” The value here is that, with knowledge of where the lower body asymmetries exist, customized workout and training regimens can be designed to strengthen and stabilize the deficient limb segments and joints aiding in coordination gain.

## 4. Discussion

Many different applications across industries exist for wearable technologies and biomechanical assessment for performance optimization and risk assessment. In the examination of applications of sports-related uses, industrial practitioners can optimize worker activities and improve worker safety by applying wearable technologies. Industries tend to use biomechanical wearables for injury mitigation during repetitive motion tasks, posture, and lifting, general task monitoring as well as ergonomic scoring of the work performed. Sports use, however, appears much broader and applies to all athletic-based loading, movements, and individual components of the movement. As stated, both sectors are concerned with injury mitigation; however, industry seems to focus on tasks more wholistically while sports break down the assessment to those individual components of movement to be studies for both improvement, risk management, and general understanding of performance.

Sensors measuring athlete biomechanical performance and risk, such as repetitive force impacts, stress and strain, and motion analysis, can help determine mitigations for industrial applications where tasks such as heavy lifting, repetitive motions, and long-term walking can impact worker health. For example, industrial athlete fatigue can be monitored like sports athletes through IMU and pressure sensors in shoe insoles. These devices can detect issues such as altered gait, asymmetries, or specific limb movements that can indicate fatigue. Studies in sports, such as the “jerk” identification in baseball and “triple flip” analysis from ice skating, also apply to the industry in the form of quickly lifting loads or scanning from different heights and awkward angles [11]. As mentioned, 3D motion capture has traditionally been required to analyze such complex motions. With integrated wearables, complex motions can be captured without significant equipment investments in a controlled environment; although, the research has shown that cost of some sensors can be a determinant for how accurate the sensors may be, such as with IMUs. This type of technology is promising for applications to jobs that occur in uncontrolled environments (e.g., outside, awkward spaces, hazardous areas) where a motion capture setup is impractical. Additionally, wearable applications that evaluate athlete return from injury, such as the concussion gait analysis, could help industry practitioners determine whether an employee is safe to return to work or if their activities harm their recovery from an injury.

Wearable applications in sports can also benefit from industry-related developments; especially when related to extensive data set analysis. The concept of Operator 4.0 would allow large amounts of real-time data collection of industry workers. By applying this type of systematic monitoring and data collection, athletic teams could be monitored in real time throughout an athletic facility for training-related activities [72]. For example, universities in the United States often have sizable collegiate football and track programs that collect data on 85 to 150 student-athletes. Using a networked-based approach such as Operator 4.0, data from each athlete could be automatically collected and used to detect trends and anomalies in personal training and team-based activities.

Some biomechanical wearable solutions are used exclusively in either sports or industry while other solutions, IMUs specifically, are used extensively in both sectors but for different task types. Table 2 provides a summary of Q1: what applications use wearable devices to improve biomechanical performance?

Likewise, this narrative summary demonstrates that biomechanical wearable sensors are being used across both sectors for the purposes of health and safety performance but with limited overlap in specific metrics being assessed. While the culture between sports and industry is very different and this speaks to a general lack adoptability on the industry [73], both sectors could stand to learn from what the other has experienced in collecting data that better aids the practitioners’ decision making. Table 3 provides conclusions about Q2: what applications use wearable devices to perform bio-mechanical risk assessment?

### Critical Issues and Problems with Wearables in Industry and Sports

While wearable technology is being promoted in the workplace and in various sports, problems with adoption and continued use remains. One of the major issues, identified for both industry and sport applications, is the “trust” factor on the wearable technology in providing accurate and meaningful data [2,48]. In industries, issues such as employee privacy, compliance, and sot-benefit ratio of the wearable devices have been identified as barriers that prevent a widespread adoption [13]. In sports, athlete’s concerns on privacy such as “big brother always watching me”, lack of customer service and challenges of dealing with wearable companies in identifying meaningful data were identified as barriers that prevent continued adoption [48]. The solution to these problems is to address these issues as a company, as a client and as a scientific community with research, development and better applications of wearable technology in industry and sports.

## 5. Conclusions

The use of wearable devices in biomechanical applications can significantly enhance human performance and refine the risk analysis processes in industrial and sports athlete applications. The narrative literature review based on articles from 2015 through 2021 revealed recent research that has been accomplished on biomechanical-capturing (and assisting) wearable devices as well as their benefits to researchers, industry analysts, and other health and safety decision-makers. Biomechanical wearables allow for performance enhancement and risk assessment in both industry and athletics by using exoskeletons and worn sensors (e.g., pressure sensors, IMU’s, and surface EMGs) to provide important data related to performance and the effects of risk-related activities on athletes and workers.

The industrial use of and risk assessment with biomechanical wearables are largely centered around injury mitigation during repetitive motion tasks, posture, and lifting, general task monitoring as well as ergonomic scoring of the work performed. Sports use of and assessment with biomechanical wearables, however, is much broader and applies to all athletic-based loading and movements as well as the individual components such as running, jumping, throwing, speed, acceleration, balance, lower body symmetry, force, pressure, impacts and more. While both sectors are focused on injury mitigation, industry seems to focus on tasks more wholistically; however, athletics are concerned with the individual components of the task. Both sectors appear to highly favor the use of IMU-based solutions and both can benefit from each other in biomechanical use cases, data analysis, and implementation. Both also suffer from the same limitations in that wearable validation must be made known against laboratory gold standards such that partitions understand and trust data outcomes. Likewise, some wearable solutions that are more precise may also be more cost prohibitive. Wearables are an ever-evolving market; taking periodic views of the current state-of-the-art will be critical for all health and safety decision makers.

## Figures and Tables

**Table 1:** Research questions and keyword search terms used in the academic research databases.

Research Question	Keywords
(Q1) What applications use wearable devices to improve biomechanical performance?	Biomechanical wearables Performance tracking wearables Wearable kinematics Workplace wearables Human performance wearables Athletic performance wearables Exoskeleton industry
(Q2) What applications use wearable devices to perform biomechanical risk assessment?	Sport risk assessment Athletic risk assessment wearables Wearables for concussion evaluation Wearable risk assessment employee Occupational wearable risk assessment

**Table 2:** Wearable biomechanical types and applications in sports and industry key takeaways.

Wearable Type	Sector/Task Type
• Exoskeletons	Industry: Upper body repetitive motion tasks Lifting Muscle load removal
• IMUs	Sports: Real-time feedback External loading Lower body joint kinetics and kinematics Running Skating Accelerations Symmetry index Kicking Jumping and flight time Throwing/pitching
	Industry: Real-time monitoring Task characterization Posture Performance analysis Indoor localization
• Pressure Sensors	Sports: Foot pressure Heel/toe strike Gait
• Surface EMG	Sports and Industry: Muscle load/activation

**Table 3:** Wearable uses for biomechanical risk assessment in sports and industry key takeaways.

Sports Risk Assessments	Industry Risk Assessments
• Running • Balance and sway • Lower body injury return rate • Lower body symmetries • Bone force and impacts • Joint rotations and movements • GRF • Speed • Surface slope • Jumping mechanics • Concussion identification • Baselining movement performance	• Fall risk • Balance loss • Gait • Repetitive motion tasks • Lifting • Ergonomic scoring

## Supplementary Materials

The following are available online at https://www.mdpi.com/article/10.3390/bioengineering9010033/s1, Figure S1: The methods followed to identify research question, search strategy and article selection, data extraction, analysis and results and reporting.
