# Supplementary material for: Wearables for Biomechanical Performance Optimization and Risk Assessment in Industrial and Sports Applications
Source: Bioengineering (Basel). 2022 Jan 13;9(1):33. doi: 10.3390/bioengineering9010033 (PMC8772827; doi:10.3390/bioengineering9010033)
Supplement: Supplementary file 1 [file bioengineering-09-00033-s001.zip › bioengineering-1510833-supplementary.pdf]

bioengineering

Review

Wearables for Biomechanical Performance Optimization and Risk Assessment in Industrial and Sports Applications

Sam McDevitt [1], Haley Hernandez [1], Jamison Hicks [2], Russell Lowell [3], Hamza Bentahaikt [4], Reuben Burch [2,5], John Ball [1,5], Harish Chander [3,5], Charles Freeman [6,*], Courtney Taylor [7] and Brock Anderson [8]

Supplementary Materials:

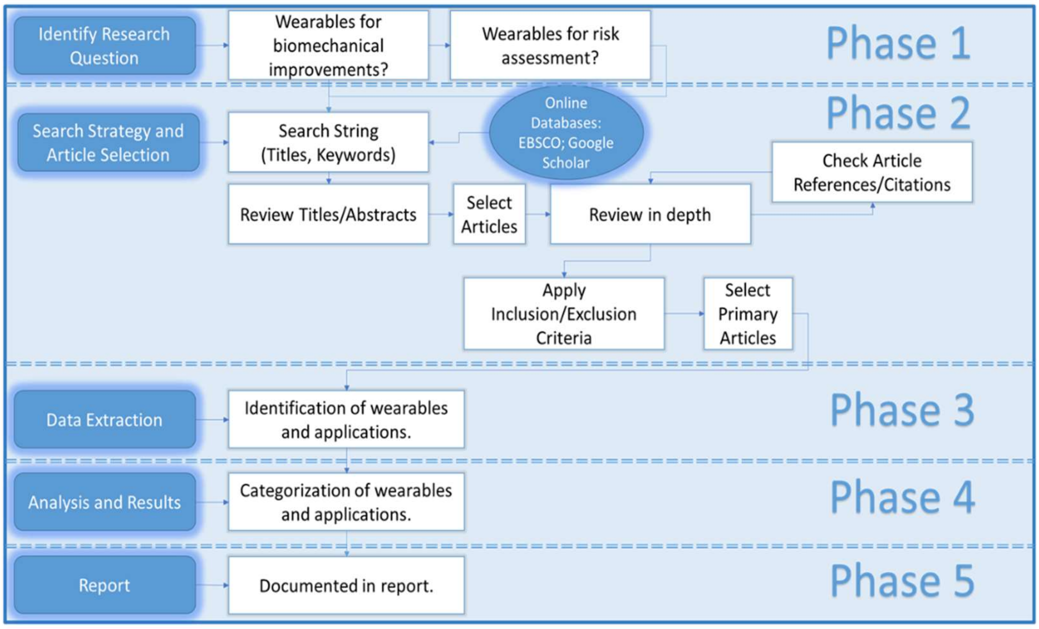

Figure S1. The methods followed to identify research question, search strategy and article selection, data extraction, analysis and results and reporting.
